# Optic nerve pit maculopathy worsened during pregnancy: a case report

**DOI:** 10.1186/s12886-020-01775-5

**Published:** 2021-01-07

**Authors:** Laura A. Torrado-Cobian, George D. Fivgas

**Affiliations:** 1 Department of Ophthalmology, Retina Division, Louisiana State University (LSU) School of Medicine, 533 Bolivar Street, Room 451B, New Orleans, LA 70112 USA; 2 LSU Department of Ophthalmology, Retina Division, Private Practice: The Retina Center, 7777 Hennessy Blvd, Suite 3000, Baton Rouge, LA 70808 USA

**Keywords:** Case report, Optic disc pit, Maculopathy, Pregnancy, Pars plana vitrectomy

## Abstract

**Background:**

To report a case of Optic Disc Pit (ODP) maculopathy exacerbated during pregnancy.

**Case presentation:**

A 30-year-old female developed unilateral blurry vision at 10-weeks gestation. Ophthalmic examination revealed left eye reduced visual acuity (VA) with the presence of subretinal fluid temporal to the disc extending to the fovea. On Spectral Domain Optical Coherence Tomography (SD-OCT) subretinal, and intraretinal fluid was confirmed. Laser photocoagulation was tried in an attempt to prevent surgical intervention without success; subsequently, pars plana vitrectomy, internal limiting membrane peel and gas tamponade was performed. Three-weeks later, a full thickness macular hole developed, and repeat surgery was performed. Nine-months after the second surgery the macular hole was closed with near complete resorption of edema.

**Conclusions:**

No trigger factors for ODP maculopathy have been reported before. We report a case of worsening ODP maculopathy during pregnancy with good visual outcome after surgical intervention.

## Background

Optic disc pit (ODP) is a rare congenital defect of the optic nerve characterized by a grayish oval depression at the optic disc, most frequently found at the inferotemporal region [1–8]. Even though maculopathy has been reported to be triggered after childbirth, there are no known factors that might precipitate or are associated with worsening optic disc maculopathy [4]. Pregnancy is known to worsen many eye disorders such as diabetic retinopathy and hypertensive retinopathy, but optic disc maculopathy has not been seen yet [9]. We therefore report a case of a pregnant young female that presented with worsening maculopathy secondary to optic disc pit.

## Case presentation

A 30-year-old female was initially referred to our clinic with a four-day history of central blurry vision in the left eye.

Her past medical history was significant for dysautonomia treated with oral fludricortisone and propranolol, and HEELP syndrome that developed during her previous pregnancy. Past ocular history was only positive for mild myopia (− 1.50 D) in both eyes. She had been followed yearly by a general ophthalmologist prior to consultation. Family history was non-contributory.

**Fig. 1:**
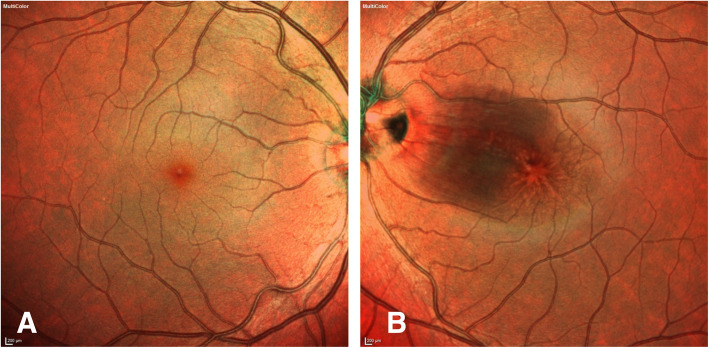
**a**. Normal fundus photo of the right eye. **b**. Fundus photo of the left eye showing temporal optic nerve pit with intraretinal fluid temporal to the disc sparing the fovea, at the first visit

**Fig. 2:**
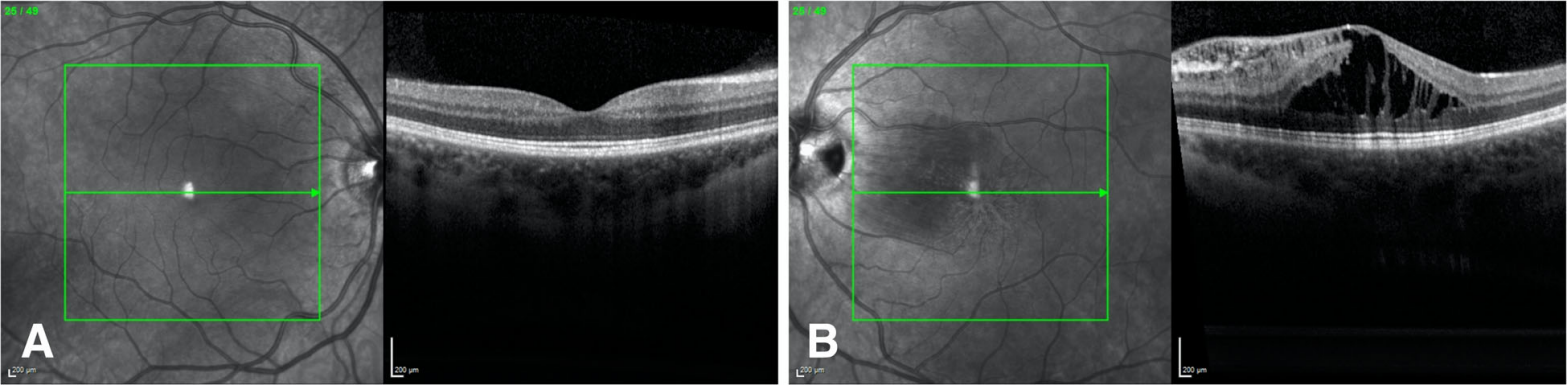
**a**. SD-OCT of the right eye with a normal foveal contour. **b**. SD-OCT of the left eye, showing blunted foveal contour, inner layer hyperreflectivity nasal to the fovea with large cysts centrally, at the first visit

**Fig. 3:**
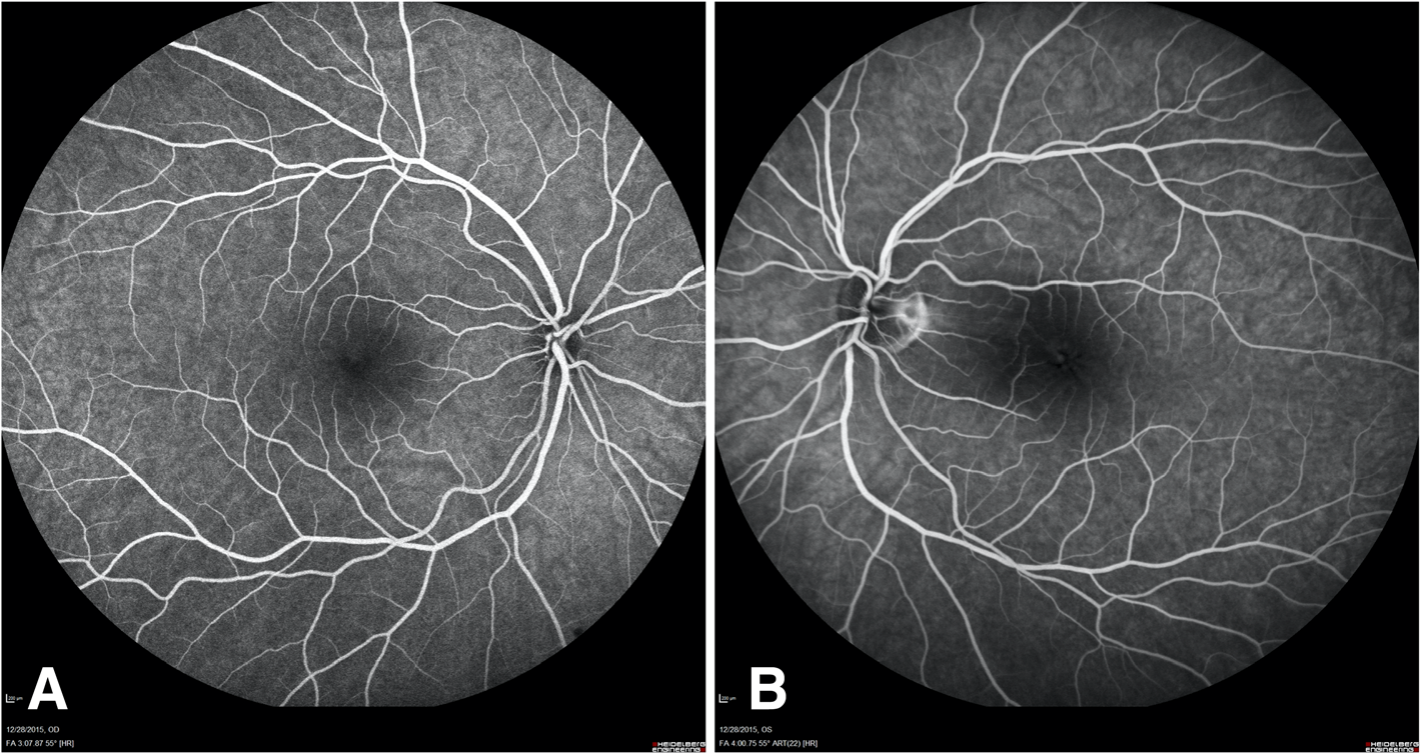
**a**. Fluorescein Angiography of the right eye showing a normal vasculature with good fill and outflow. **b**. Fluorescein Angiography of the left eye showing hyperfluorescence spots temporal to the optic nerve, at the first visit

Her exam revealed a best corrected visual acuity (BCVA) of 20/25 in the right eye and 20/30 in the left eye. Intraocular pressure (IOP) was 16 mm of Hg in both eyes. Her external ocular exam, anterior segment and vitreous were normal bilaterally. Retinal exam in the right eye was unremarkable, and in the left eye an optic nerve pit was noted with the presence of intraretinal fluid temporal to the disc, sparing the fovea. (Fig. 1a and b) Spectral Domain Optical Coherence Tomography (SD-OCT) showed the right eye with a normal foveal contour and the left eye with a blunted foveal contour, inner layer hyperreflectivity nasal to the fovea, and large cystoid spaces centrally. (Fig. 2a and b) Fluorescein Angiography (FA) revealed a normal vasculature with good fill and outflow in the right eye and the left eye with hyperfluorescent spots temporal to the optic nerve. (Fig. 3a and b) Since her visual acuity was good and appeared stable on review of old records and anatomically there was no macular detachment, the decision was made to monitor.

**Fig. 4:**
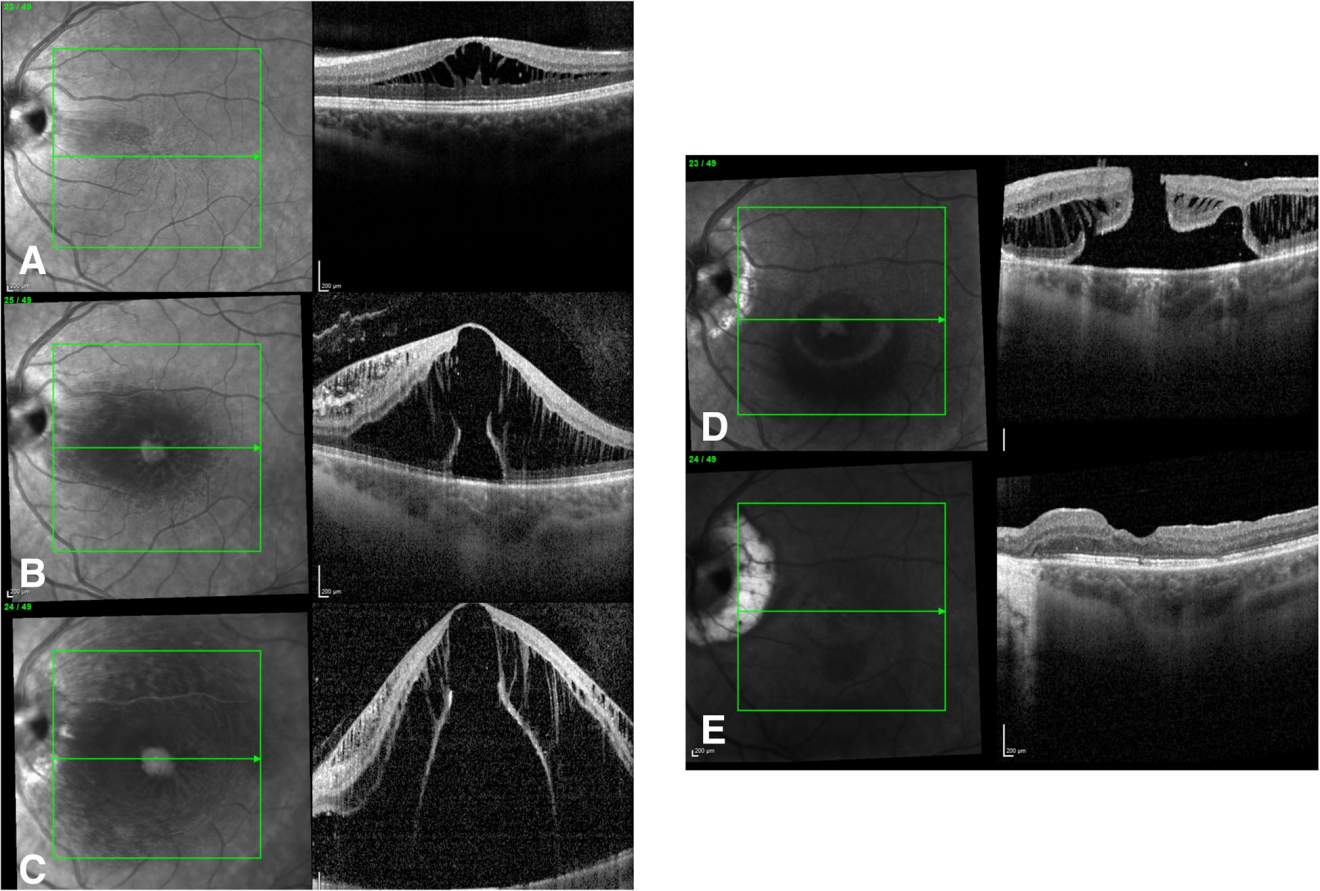
**a**. SD-OCT of the left eye, showing worsening of sub-foveal intraretinal and subretinal fluid, after pregnancy diagnosis. **b**. SD-OCT of the left eye, showing worsening of sub-foveal intraretinal and subretinal fluid, after three-week observation. **c**. SD-OCT of the left eye, one-month after laser treatment. Edema continued to progress and large outer macular defect along with a thin tissue of inner layer bridging the defect. **d**. SD-OCT of the left eye showing a full thickness macular hole three-week after intervention for OPD maculopathy. **e**. SD-OCT of the left eye, nine months after second surgery for full thickness macular hole

Two years later, after remaining stable during follow-up, the patient presented to the clinic with a 3-day history of decreased vision. Interval history was significant for pregnancy of 10-weeks gestation. On examination BCVA was 20/25 and 20/60 in the right and left eye respectively, and IOP of 16 mmHg in both eyes. Right eye examination was still unremarkable. Left eye retinal exam disclosed subretinal and intraretinal fluid from the optic nerve pit extending to the fovea. SD-OCT confirmed the presence of sub-foveal, intraretinal (IR) and subretinal (SR) fluid. (Fig. 4a) Due to pregnancy, close observation was recommended. The patient was followed-up weekly for the following three weeks and IR and SR fluid continued to worsen. (Fig. 4b) Since she was losing vision, treatment was recommended and laser photocoagulation was deemed to be the least stressful option. Thermal laser photocoagulation at the temporal edge of the pit was performed in an attempt to stop the edema from progressing. One-month after laser treatment, the edema continued to progress and concurrently vitreomacular traction was noted on SD-OCT (Fig. 4c). At this point, the patient was only 19 weeks pregnant; after discussion with her OB-GYN and maternal fetal medicine specialist, pars plana vitrectomy (PPV) with internal limiting membrane (ILM) peel was planned under local anesthesia. Surgery was uneventful and there was some anatomic improvement, but 3-weeks after surgery a full-thickness macular hole (FTMH) (Fig. 4d) developed. As such, at 24-weeks gestation vitrectomy was repeated by extending ILM peel temporally and out of arcades in an attempt to further relieve tangential traction and give more pliability to allow for macular hole edge apposition. Nine-months after surgery, macular hole closure was achieved and the patient continued to improve with BCVA at last follow-up of 20/20 in the right eye and 20/50 in the left eye (Fig. 4e).

## Discussion

Maculopathy is a well described condition occurring in 25–75% of patients with ODP and being responsible for significant vision loss in up to 65% of the patients [1, 4, 9–11]. Macular changes described are serous detachment, cystoid degeneration, degenerative pigmentary changes and macular schisis [1, 6–8, 12]. Even though some reports describe spontaneous resolution in about 25% of patients, the majority have a poor visual prognosis with 80% of patients reporting a visual acuity of 20/200 or worse [4, 6, 7, 13].

The exact mechanism of maculopathy development is still unknown; the vitreous, cerebrospinal fluid, leakage from blood vessels and fluid from choroid through Bruch’s membrane are four possible origins that have been postulated [2–4, 7, 11, 14, 15].

There is no universally accepted management, but the current standard treatment of ODP maculopathy is PPV, with posterior vitreous detachment induction, ILM peeling, peripapillary laser, fluid-air exchange and gas tamponade [5, 11, 13]. Other new innovative techniques described to seal the ODP include inverting peeled ILM into the ODP, ILM chunk transplantation, Tissel fibrin sealant, autologous scleral tissue flap and autologous platelet injection on the optic pit [7, 10, 13, 16].

Laser photocoagulation at the temporal disc margins was introduced as a conservative treatment to prevent further fluid accumulation by creating a barrier between the ODP and subretinal space [5, 7, 11]. Even though results and time for improvement after laser treatment are unclear, it was a suitable option for our patient, taking into consideration that BCVA and ODP maculopathy were worsening in the presence of an early pregnancy.

FTMH has been associated with chronic macular detachment, leading to irreversible visual loss [5, 11]. Many techniques have been described to prevent a FTMH from happening such as vitrectomy alone, vitrectomy and PVD induction and ILM peeling but none of them have shown any improvement in anatomy or visual acuity [17, 18]. Taking into consideration that FTMH has been reported in up to 57% of the cases and has been attributed to peeling over thinned-out retina as previously described by Shukla and co-authors [1, 7, 17], we might question if ILM peeling is necessary. However, ILM peeling relieves the tangential traction needed to prevent the passage of fluid from the ODP to the macula [17]. Recently, the use of platelet-rich plasma for recurrent ODP maculopathy and FTMH has been described with promising results but further studies are needed to prove its efficacy and safetuy [18]. Our patient had a good outcome after repeat PPV with extended ILM peeling and gas tamponade, achieving FTMH closure and improvement in VA, which shows that a careful removal of the posterior hyaloid with complete release of the tangential and anteroposterior traction is very important when performing surgery in a thinned-out retina.

## Conclusion

To date, there are no known triggers for developing ODP maculopathy. There is one case report which describes childbirth as a trigger to developing ODP maculopathy due to differences between intraocular pressure and intracranial pressure, but nevertheless our patient presented with worsening ODP maculopathy during pregnancy. The relationship between pregnancy and childbirth and ODP maculopathy progression is unknown but might be related to hormones or Valsalva phenomenon present during this period of time. Moreover, poor visual prognosis is a common finding in these patients especially after FTMH formation. We present a case with a good visual outcome after surgical intervention under local anesthesia during pregnancy. Despite a severely edematous macula and thinned fovea leading to a full thickness macular hole after the initial surgery, a second surgery proved successful in closure of the macular hole and substantial visual improvement. Surgery performed under local anesthesia with consent from the attending obstetrician and maternal fetal medicine specialist may be a successful route to stabilize, improve, and spare vision loss in this susceptible population.

## Data Availability

The datasets generated and/or analysed during the current study are not publicly available due to protection of the patient’s personal information but are available from the corresponding author on reasonable request.

## Abbreviations

ODP: Optic disc pit maculopathy
VA: Visual acuity
SD-OCT: Spectral domain optical coherence tomography
BCVA: Best-corrected visual acuity
IOP: Intraocular pressure
FA: Fluorescein angiography
IR: Intraretinal
SR: Subretinal
PPV: Pars plana vitrectomy
ILM: Internal limiting membrane
FTMH: Full-thickness macular hole
OB-GYN: Obstetrician and Gynecologyst
